# A Review of Modeling of Composite Structures †

**DOI:** 10.3390/ma17020446

**Published:** 2024-01-17

**Authors:** Wenbin Yu

**Affiliations:** School of Aeronautics and Astronautics, Purdue University, West Lafayette, IN 47906, USA; wenbinyu@purdue.edu

**Keywords:** modeling of composite structures, axiomatic method, variational asymptotic method, formal asymptotic method, constitutive modeling

## Abstract

This paper provides a brief review on modeling of composite structures. Composite structures in this paper refer to any structure featuring anisotropy and heterogeneity, including but not limited to their traditional meaning of composite laminates made of unidirectional fiber-reinforced composites. Common methods used in modeling of composite structures, including the axiomatic method, the formal asymptotic method, and the variational asymptotic method, are illustrated in deriving the classical lamination theory for the composite laminated plates. Future research directions for modeling composite structures are also pointed out.

## 1. Introduction

**Figure 1:**
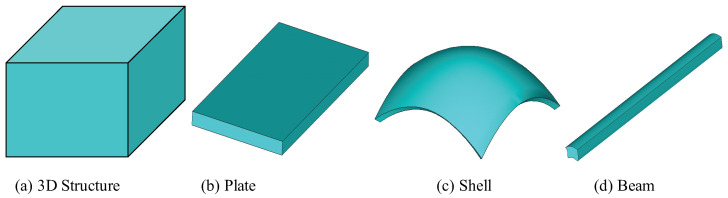
Typical structural components [1].

We usually classify structures in terms of their external geometry. If all three dimensions of a structural component are of similar size, it is a three-dimensional (3D) solid (Figure 1a). If one dimension of a structural component is much smaller than the other two dimensions, it is a plate (Figure 1b) or a shell (Figure 1c), depending on whether the undeformed in-plane shape is flat or curved, respectively. The small dimension is commonly called the thickness. If two dimensions of a structural component are much smaller than the third dimension, it is a beam (Figure 1d). Usually the large dimension is called the axis of the beam and a reference line can be defined for the beam along the large dimension. The two small dimensions are commonly called the cross-section for typical beam-like structures. The reference line can be as general as a spatial curve, which is the case for initially curved and twisted beams. If the beam itself is made of thin-walled components, that is, the wall thickness is much smaller than the cross-sectional size, it is a thin-walled beam. We can collectively denote plates, shells, beams, and thin-walled beams as dimensionally reducible structures, emphasizing the fact that one or more small dimensions can be eliminated for developing a simplified structural model with reduced dimensions for adequate prediction of their behaviors.

As far as modeling is concerned, this classification only considers the external geometry, whereas the internal construction of these structures can be arbitrary. For example, a sandwich flat panel with a honeycomb core can be modeled as a plate and a high-aspect-ratio wing of aircraft can be modeled as a beam without clearly defined cross-sections. In this sense, all engineering structural systems, despite their complexity, can be considered as formed by a combination of structural components in terms of three-dimensional (3D) structures, two-dimensional (2D) plates/shells, and/or one-dimensional (1D) beams, as shown in Figure 1 with possible complex internal constructions with general anisotropic materials.

Recent decades have witnessed great advances in materials technology and manufacturing techniques. Structures traditionally made of isotropic homogeneous materials are now increasingly made of advanced materials featuring anisotropy and heterogeneity. Such structures are called composite structures in this paper. Since, in the literature, composite structures usually refer to laminated structures made of unidirectional fiber-reinforced composites (UDFRCs), it is emphasized that composite structures in this paper refer to structures featuring anisotropy and heterogeneity at the continuum scale (the length scale of micron and larger), such as laminates, sandwich structures, stiffened structures, rotor blades made of UDFRCs, tow-steered composites, short fiber composites, particle-reinforced composites, woven composites, metamaterials, etc. Cutting-edge manufacturing techniques can routinely manipulate geometry and material at the continuum scale. It is conventionally believed that the classical models (Cauchy continuum model for 3D solids, Kirchhoff–Love model or Reissner–Mindlin model for plates/shells, Euler–Bernoulli model or Timoshenko model for regular beams, Vlasov model for thin-walled beams) developed for structures made of isotropic homogeneous materials several centuries ago are not applicable for composite structures made of anisotropic and/or heterogeneous materials. This motivated many non-classical models such as the Cosserat solid model, high-order, zig-zag, or layerwise plate/shell models, or many advanced beam models developed to tackle the complexity of composite structures introduced by anisotropy and heterogeneity of advanced materials and arbitrary internal structural constructions. Although these non-classical models provide better predictions than the classical models for some cases, it is not easy to implement them in the existing structural solvers, including general-purpose finite element analysis (FEA) packages such as Abaqus, Ansys, Nastran, or special-purpose comprehensive analysis codes such as RCAS (Rotorcraft Comprehensive Analysis System). Instead, special-purpose finite element codes must be developed. These codes cannot be easily incorporated into commercial finite element codes due to their complexity and limited uses. Thus, these non-classical models mainly have academic value and are not used by practitioners very much.

To leverage existing structural solvers provided by off-the-shelf commercial finite element packages, we have to find a way to construct models suitable for composite structures featuring complex internal constructions and made of advanced anisotropic heterogeneous materials, while at the same time being compatible with those available in existing structural solvers. This can be achieved by recognizing the fact that each model can be separated into three parts, including kinematics (displacements, strains, and the corresponding strain–displacement relations, and compatibility equations), kinetics (stresses, forces, moments, and the corresponding equations of motion or equilibrium equations), and energetics (constitutive relations). Both kinematics and kinetics are implemented in existing structural solvers, while the constitutive relations can be obtained externally by constitutive modeling and used as inputs for existing structural solvers. Now, instead of constantly developing better non-classical models for composite structures, the challenge can be met by developing better constitutive modeling to bridge the original model and a desirable classical structural model so that the loss of accuracy between these two model representations of the same structure can be minimized. The constitutive modeling was not part of the traditional paradigm of structural mechanics, as traditional structural mechanics theories do not clearly separate constitutive relations from kinematics and kinetics, while it is a distinctive feature of the work of Hodges and his collaborators [2].

Many reviews with an extensive bibliography on modeling of composite structures are available in the literature (Grigolyuk et al. [3] with 161 references, Leissa [4] with 352 references, Grigolyuk et al. [5] with 74 references, Kapania et al. [6] with 145 references, Noor [7] with 203 references, Carrera [8] with 325 references, Carrera [9] with 138 references, Khandan et al. [10] with 159 references, Liew et al. [11] with 247 references, Li [12] with 261 references, Carrera et al. [13] with 168 references). This paper will not repeat these efforts. Instead we will first provide a brief introduction to the classical models, then we will review three main methods used to derive the Kirchhoff–Love model for composite laminated plates. Finally, we will point out the future challenges and research directions related to modeling composite structures.

## 2. Introduction to Classical Structural Models

In this section, we introduce the kinemics, kinetics, and constitutive relations of the classical structural models commonly used in engineering, including the Cauchy continuum model, the Kirchhoff–Love plate/shell model, the Reissner–Mindlin plate/shell model, the Euler–Bernoulli beam model, the Timoshenko beam model, and the Vlasov beam model. Since this paper focuses on the methodology on structural modeling, the process of reducing the original 3D problem into a 1D model for beams and 2D model for plates/shells, only the final model form in terms of kinematics, equilibrium equations, and constitutive relations is presented here, without details of how these equations are obtained. Even if historically the models are derived very differently, for example, the Reissner model and the Mindlin model, as long as the final form remains the same, we collectively call their model the Reissner–Mindlin model. Furthermore, this paper is not interested in how to solve these models for specific static or dynamic behavior. Instead, we only focus on contrasting the main methods used to derive these models.

For the purpose of illustrating the modeling method, we restrict this introduction to linear elastic behavior. We use $x_{1},x_{2},x_{3}$ to denote the coordinate systems. In particular, for beams, $x_{1}$ is along the beam reference line, and $x_{2}$ and $x_{3}$ describe the cross-sectional plane; for plates/shells, $x_{1}$ and $x_{2}$ describe the reference surface, while $x_{3}$ is along the transverse normal.

### 2.1. Cauchy Continuum Model

The kinematics of the Cauchy continuum model contains three displacements $u_{1},u_{2},u_{3}$ and six strains $\varepsilon_{11},\varepsilon_{22},\varepsilon_{33},\varepsilon_{23},\varepsilon_{13},\varepsilon_{12}$. The infinitesimal strains are defined in terms of the displacements as

(1) $$\begin{array}{cc} \varepsilon_{11} & =\frac{\partial u_{1}}{\partial x_{1}},\;\varepsilon_{22}=\frac{\partial u_{2}}{\partial x_{2}},\;\varepsilon_{33}=\frac{\partial u_{3}}{\partial x_{3}}\\ 2\varepsilon_{23} & =\frac{\partial u_{2}}{\partial x_{3}}+\frac{\partial u_{3}}{\partial x_{2}},\;2\varepsilon_{13}=\frac{\partial u_{1}}{\partial x_{3}}+\frac{\partial u_{3}}{\partial x_{1}},\;2\varepsilon_{12}=\frac{\partial u_{1}}{\partial x_{2}}+\frac{\partial u_{2}}{\partial x_{1}} \end{array}$$

The kinetic variables of the Cauchy continuum model are six stresses $\sigma_{11},\sigma_{22},\sigma_{33}$, $\sigma_{23}$, $\sigma_{13},\sigma_{12}$. If Cauchy stress tensor is used, these stresses are governed by the following equations of equilibrium:

(2) $$\begin{array}{cc} \frac{\partial \sigma_{11}}{\partial x_{1}}+\frac{\partial \sigma_{12}}{\partial x_{2}}+\frac{\partial \sigma_{13}}{\partial x_{3}}+f_{1} & =0\\ \frac{\partial \sigma_{12}}{\partial x_{1}}+\frac{\partial \sigma_{22}}{\partial x_{2}}+\frac{\partial \sigma_{23}}{\partial x_{3}}+f_{2} & =0\\ \frac{\partial \sigma_{13}}{\partial x_{1}}+\frac{\partial \sigma_{23}}{\partial x_{2}}+\frac{\partial \sigma_{33}}{\partial x_{3}}+f_{3} & =0 \end{array}$$

where $f_{1},f_{2},f_{3}$ are distributed body forces per unit volume in three directions. The constitutive relations of the Cauchy continuum model for the linear elastic behavior are described using the generalized Hooke’s law as

(3) $$\left\{\begin{array}{c} \sigma_{11}\\ \sigma_{22}\\ \sigma_{33}\\ \sigma_{23}\\ \sigma_{13}\\ \sigma_{12} \end{array}\right\}=\left[\begin{array}{cccccc} C_{11} & C_{12} & C_{13} & C_{14} & C_{15} & C_{16}\\ C_{12} & C_{22} & C_{23} & C_{24} & C_{25} & C_{26}\\ C_{13} & C_{23} & C_{33} & C_{34} & C_{35} & C_{36}\\ C_{14} & C_{24} & C_{34} & C_{44} & C_{45} & C_{46}\\ C_{15} & C_{25} & C_{35} & C_{45} & C_{55} & C_{56}\\ C_{16} & C_{26} & C_{36} & C_{46} & C_{56} & C_{66} \end{array}\right]\left\{\begin{array}{c} \varepsilon_{11}\\ \varepsilon_{22}\\ \varepsilon_{33}\\ 2\varepsilon_{23}\\ 2\varepsilon_{13}\\ 2\varepsilon_{12} \end{array}\right\}$$

The 6×6 symmetric matrix is the stiffness matrix, and its inverse is the compliance matrix.

(4) $$\left\{\begin{array}{c} \varepsilon_{11}\\ \varepsilon_{22}\\ \varepsilon_{33}\\ 2\varepsilon_{23}\\ 2\varepsilon_{13}\\ 2\varepsilon_{12} \end{array}\right\}=\left[\begin{array}{cccccc} S_{11} & S_{12} & S_{13} & S_{14} & S_{15} & S_{16}\\ S_{12} & S_{22} & S_{23} & S_{24} & S_{25} & S_{26}\\ S_{13} & S_{23} & S_{33} & S_{34} & S_{35} & S_{36}\\ S_{14} & S_{24} & S_{34} & S_{44} & S_{45} & S_{46}\\ S_{15} & S_{25} & S_{35} & S_{45} & S_{55} & S_{56}\\ S_{16} & S_{26} & S_{36} & S_{46} & S_{56} & S_{66} \end{array}\right]\left\{\begin{array}{c} \sigma_{11}\\ \sigma_{22}\\ \sigma_{33}\\ \sigma_{23}\\ \sigma_{13}\\ \sigma_{12} \end{array}\right\}$$

Material properties are usually measured in the material coordinate system, which implies that we need to express constitutive relations in the material coordinate system first. However, the kinematics and kinetics are usually formulated in the global coordinate system. A proper transformation according to the tensorial transformation laws is needed to transfer the constitutive relations into the global coordinate system.

For isotropic materials, the constitutive relations can be expressed in terms of Young’s modulus *E* and Poisson’s ratio ν as

$$\begin{array}{cc} \; & C_{11}=C_{22}=C_{33}=\frac{E(1-\nu )}{\left(1+\nu )(1-2\nu \right)}\\ \; & C_{12}=C_{13}=C_{23}=\frac{E\nu }{\left(1+\nu )(1-2\nu \right)}\\ \; & C_{44}=C_{55}=C_{66}=\frac{E}{2(1+\nu )} \end{array}$$

and all other terms in the stiffness matrix of Equation (3) are zero.

Equations (1)–(3) form a system of 15 equations underpinning the Cauchy continuum model to be solved along with appropriate boundary conditions for 15 unknowns (three displacements, six strains, and six stresses, all of which are functions of three coordinates $x_{1},x_{2},x_{3}$ used to describe the 3D body). Kinematics and kinetics remain the same no matter whether the structure is made of isotropic homogeneous materials such as metals or anisotropic heterogeneous materials such as composites. This model has been implemented in many FEA codes which have 3D solid elements.

### 2.2. Kirchhoff–Love Plate/Shell Model

Kirchhoff originally developed the classical plate model for flat panels based on a set of ad hoc assumptions, including the transverse normal (a material line along the thickness direction) being rigid in the thickness direction, remaining straight and perpendicular to the reference surface, and the structure experiencing a plane stress state, during the deformation. Love later extended the same set of assumptions to curved panels to develop the classical shell model. Since both models are based on the same set of assumptions and take the same functional form, we collectively call them the Kirchhoff–Love model. Since the Kirchhoff–Love model is the first complete model developed in history, it is also called the classical plate/shell model. Here, for simplicity, we use the classical plate model for illustrative purposes. The kinematics of the Kirchhoff–Love plate model contains three displacements ${\bar{u}}_{1},{\bar{u}}_{2},{\bar{u}}_{3}$, three in-plane strains $\epsilon_{11},\epsilon_{22},\epsilon_{12}$, and three curvatures $\kappa_{11},\kappa_{22},\kappa_{12}$. It is noted that most traditional formulations of the classical plate model only include the bending behavior (one displacement ${\bar{u}}_{3}$ and three curvatures $\kappa_{11},\kappa_{22},\kappa_{12}$). Here, for the connection to composite structures, we also include the in-plane behavior in the formulation.

Specifically for a plate, the strain–displacement relations are given as

(5) $$\begin{array}{cc} \epsilon_{11} & =\frac{\partial {\bar{u}}_{1}}{\partial x_{1}},\;\epsilon_{22}=\frac{\partial {\bar{u}}_{2}}{\partial x_{2}},\;2\epsilon_{12}=\frac{\partial {\bar{u}}_{1}}{\partial x_{2}}+\frac{\partial {\bar{u}}_{2}}{\partial x_{1}}\\ \kappa_{11} & =-\frac{\partial^{2}{\bar{u}}_{3}}{\partial x_{1}^{2}},\;\kappa_{22}=-\frac{\partial^{2}{\bar{u}}_{3}}{\partial x_{2}^{2}},\;\kappa_{12}=-\frac{\partial^{2}{\bar{u}}_{3}}{\partial x_{1}\partial x_{2}} \end{array}$$

The kinetic variables of the Kirchhoff–Love model contain three in-plane forces $N_{11},N_{22},N_{12}$ and three moments $M_{11},M_{22},M_{12}$. These kinetic variables are governed by the following three equations of equilibrium:

(6) $$\begin{array}{cc} \; & \frac{\partial N_{11}}{\partial x_{1}}+\frac{\partial N_{12}}{\partial x_{2}}+p_{1}=0\\ \; & \frac{\partial N_{21}}{\partial x_{1}}+\frac{\partial N_{22}}{\partial x_{2}}+p_{2}=0\\ \; & \frac{\partial^{2}M_{11}}{\partial {x_{1}}^{2}}+\frac{\partial^{2}M_{22}}{\partial {x_{2}}^{2}}+2\frac{\partial^{2}M_{12}}{\partial x_{1}\partial x_{2}}+\frac{\partial q_{2}}{\partial x_{1}}-\frac{\partial q_{1}}{\partial x_{2}}+p_{3}=0 \end{array}$$

where $p_{1},p_{2},p_{3}$ are equivalent forces in three directions and $q_{1},q_{2}$ are equivalent moments in two in-plane directions distributed over the reference surface. It is noted here that most traditional formulations of the classical plate model only include the transverse pressure $p_{3}$ as the distributed force. In fact, distributed forces could exist in three directions and distributed bending moment could exist in two directions. These loads can be rigorously derived from the distributed body forces, tractions on the top and bottom surfaces, as shown in Equation (33).

The constitutive relations of the Kirchhoff–Love model can be expressed using the following six equations:

(7) $$\left\{\begin{array}{c} N_{11}\\ N_{22}\\ N_{12}\\ M_{11}\\ M_{22}\\ M_{12} \end{array}\right\}=\left[\begin{array}{cccccc} A_{11} & A_{12} & A_{16} & B_{11} & B_{12} & B_{16}\\ A_{12} & A_{22} & A_{26} & B_{21} & B_{22} & B_{26}\\ A_{16} & A_{26} & A_{66} & B_{61} & B_{62} & B_{66}\\ B_{11} & B_{21} & B_{61} & D_{11} & D_{12} & D_{16}\\ B_{12} & B_{22} & B_{62} & D_{12} & D_{22} & D_{26}\\ B_{16} & B_{26} & B_{66} & D_{16} & D_{26} & D_{66} \end{array}\right]\left\{\begin{array}{c} \epsilon_{11}\\ \epsilon_{22}\\ 2\epsilon_{12}\\ \kappa_{11}\\ \kappa_{22}\\ 2\kappa_{12} \end{array}\right\}$$

This 6×6 symmetric matrix is the stiffness matrix and its inverse is the compliance matrix for the classical plate model.

(8) $$\left\{\begin{array}{c} \epsilon_{11}\\ \epsilon_{22}\\ 2\epsilon_{12}\\ \kappa_{11}\\ \kappa_{22}\\ 2\kappa_{12} \end{array}\right\}={\left[\begin{array}{cccccc} A_{11} & A_{12} & A_{16} & B_{11} & B_{12} & B_{16}\\ A_{12} & A_{22} & A_{26} & B_{21} & B_{22} & B_{26}\\ A_{16} & A_{26} & A_{66} & B_{61} & B_{62} & B_{66}\\ B_{11} & B_{21} & B_{61} & D_{11} & D_{12} & D_{16}\\ B_{12} & B_{22} & B_{62} & D_{12} & D_{22} & D_{26}\\ B_{16} & B_{26} & B_{66} & D_{16} & D_{26} & D_{66} \end{array}\right]}^{-1}\left\{\begin{array}{c} N_{11}\\ N_{22}\\ N_{12}\\ M_{11}\\ M_{22}\\ M_{12} \end{array}\right\}$$

We used the conventional notation, A,B,D matrices, well-known in the classical lamination theory (CLT). One difference is noted here. *B* is known to be symmetric in CLT due to the symmetry of the plane stress reduced stiffness matrix *Q* (i.e., $B_{21}=B_{12},B_{61}=B_{16},B_{62}=B_{26}$. However, it is not symmetric for a general anisotropic, heterogeneous flat panel to be modeled using the classical plate model. Thus, *B* used in Equations (7) and (8) allows the possibility of being unsymmetric.

If a homogeneous plate is made of a single isotropic material, and the origin of $x_{3}$ is chosen to be at the center of the thickness, the constitutive relations are

(9) $$\begin{array}{cc} \; & \left\{\begin{array}{c} N_{11}\\ N_{22}\\ N_{12} \end{array}\right\}=\frac{Eh}{1-\nu^{2}}\left[\begin{array}{ccc} 1 & \nu & 0\\ \nu & 1 & 0\\ 0 & 0 & \frac{1-\nu }{2} \end{array}\right]\left\{\begin{array}{c} \epsilon_{11}\\ \epsilon_{22}\\ 2\epsilon_{12} \end{array}\right\}\\ \; & \left\{\begin{array}{c} M_{11}\\ M_{22}\\ M_{12} \end{array}\right\}=\frac{Eh^{3}}{12(1-\nu^{2})}\left[\begin{array}{ccc} 1 & \nu & 0\\ \nu & 1 & 0\\ 0 & 0 & \frac{1-\nu }{2} \end{array}\right]\left\{\begin{array}{c} \kappa_{11}\\ \kappa_{22}\\ 2\kappa_{12} \end{array}\right\} \end{array}$$

Equations (5)–(7) form a system of 15 equations underpinning the Kirchhoff–Love model to be solved along with appropriate boundary conditions for 15 unknowns (three displacements, six strain variables, and six stress resultants, all of which are functions of $x_{1}$ and $x_{2}$ describing the two-dimensional (2D) reference surface). Kinematics and kinetics remain the same no matter whether the structure is made of isotropic homogeneous materials such as metals or anisotropic heterogeneous materials such as composites. The only difference is that the plate/shell stiffness matrix in Equation (7) could be fully populated if the plate/shell is made of composites. This model can be applied to model flat panels with arbitrary heterogeneity (e.g., laminates, sandwich panels with honeycomb cores, corrugated panels) as long as the thickness remains relatively small compared to the in-plane dimensions. The Kirchhoff–Love model governed by these equations has been implemented in many FEA codes which have plate/shell elements.

It is noted that although the Kirchhoff–Love model was originally developed based on a set of a priori assumptions as aforementioned, such assumptions are not absolutely needed to derive this model. In fact, one can use a more advanced modeling technique such as the variational asymptotic method (VAM) [14] to be illustrated later. Thus, the Kirchhoff–Love model presented here only refers to the model which has 15 field variables of $x_{1},x_{2}$ governed by the 15 equations in Equations (5)–(7). In other words, the thickness could be deformed, not necessarily perpendicular to the reference surface, and all six stress components including both in-plane stresses and transverse stresses could exist.

### 2.3. Reissner–Mindlin Plate/Shell Model

When the thickness of the panel is not very small compared to the in-plane dimensions, or when the deformation cannot be fully captured using in-plane strains ($\epsilon_{11},\epsilon_{22},\epsilon_{12}$) and the curvatures ($\kappa_{11},\kappa_{22},\kappa_{12}$), the Kirchhoff–Love model is inadequate and a refined model is needed. A refined plate/shell model beyond the Kirchhoff–Love model is the Reissner–Mindlin model, so named due to independent contributions of Reissner and Mindlin to its development. The kinematics of the Reissner–Mindlin model contains five displacement variables, including three displacements ${\bar{u}}_{1},{\bar{u}}_{2},{\bar{u}}_{3}$ and two rotations $\theta_{1},\theta_{2}$. This model features three in-plane strains $\epsilon_{11},\epsilon_{22},\epsilon_{12}$, three curvatures $\kappa_{11},\kappa_{22},\kappa_{12}$, and two transverse shear strains $\epsilon_{13},\epsilon_{23}$. For a plate, the strain–displacement relations are given as

(10) $$\begin{array}{cc} \epsilon_{11} & =\frac{\partial {\bar{u}}_{1}}{\partial x_{1}},\;\epsilon_{22}=\frac{\partial {\bar{u}}_{2}}{\partial x_{2}},\;2\epsilon_{12}=\frac{\partial {\bar{u}}_{1}}{\partial x_{2}}+\frac{\partial {\bar{u}}_{2}}{\partial x_{1}}\\ \kappa_{11} & =\frac{\partial \theta_{2}}{\partial x_{1}},\;\kappa_{22}=-\frac{\partial \theta_{1}}{\partial x_{2}},\;2\kappa_{12}=\frac{\partial \theta_{2}}{\partial x_{2}}-\frac{\partial \theta_{1}}{\partial x_{1}}\\ 2\epsilon_{13} & =\frac{\partial {\bar{u}}_{3}}{\partial x_{1}}+\theta_{2},\;2\epsilon_{23}=\frac{\partial {\bar{u}}_{3}}{\partial x_{2}}-\theta_{1} \end{array}$$

The kinetic variables of the Reissner–Mindlin model contain in-plane forces $N_{11},N_{22},N_{12}$, moments $M_{11},M_{22},M_{12}$, and transverse shear forces $N_{13},N_{23}$. They are governed by the following five equations of equilibrium:

(11) $$\begin{array}{cc} \; & \frac{\partial N_{11}}{\partial x_{1}}+\frac{\partial N_{12}}{\partial x_{2}}+p_{1}=0\\ \; & \frac{\partial N_{21}}{\partial x_{1}}+\frac{\partial N_{22}}{\partial x_{2}}+p_{2}=0\\ \; & \frac{\partial N_{13}}{\partial x_{1}}+\frac{\partial N_{23}}{\partial x_{2}}+p_{3}=0\\ \; & \frac{\partial M_{12}}{\partial x_{1}}+\frac{\partial M_{22}}{\partial x_{2}}-q_{1}-N_{23}=0\\ \; & \frac{\partial M_{11}}{\partial x_{1}}+\frac{\partial M_{21}}{\partial x_{2}}+q_{2}-N_{13}=0 \end{array}$$

The constitutive relations of the Reissner–Mindlin model can be expressed using the following eight equations:

(12) $$\left\{\begin{array}{c} N_{11}\\ N_{22}\\ N_{12}\\ M_{11}\\ M_{22}\\ M_{12}\\ N_{13}\\ N_{23} \end{array}\right\}=\left[\begin{array}{cccccccc} A_{11} & A_{12} & A_{16} & B_{11} & B_{12} & B_{16} & Y_{11} & Y_{12}\\ A_{12} & A_{22} & A_{26} & B_{21} & B_{22} & B_{26} & Y_{21} & Y_{22}\\ A_{16} & A_{26} & A_{66} & B_{61} & B_{62} & B_{66} & Y_{31} & Y_{32}\\ B_{11} & B_{21} & B_{61} & D_{11} & D_{12} & D_{16} & Y_{41} & Y_{42}\\ B_{12} & B_{22} & B_{62} & D_{12} & D_{22} & D_{26} & Y_{51} & Y_{52}\\ B_{16} & B_{26} & B_{66} & D_{16} & D_{26} & D_{66} & Y_{61} & Y_{62}\\ Y_{11} & Y_{21} & Y_{31} & Y_{41} & Y_{51} & Y_{61} & G_{11} & G_{12}\\ Y_{12} & Y_{22} & Y_{32} & Y_{42} & Y_{52} & Y_{62} & G_{12} & G_{22} \end{array}\right]\left\{\begin{array}{c} \epsilon_{11}\\ \epsilon_{22}\\ 2\epsilon_{12}\\ \kappa_{11}\\ \kappa_{22}\\ 2\kappa_{12}\\ 2\epsilon_{13}\\ 2\epsilon_{23} \end{array}\right\}$$

This 8×8 symmetric matrix is the stiffness matrix and its inverse is the compliance matrix for the Reissner–Mindlin model. $G_{ij}$ (i=1,2;j=1,2) denote the transverse shear stiffness terms and $Y_{ij}$ (i=1,…,6;j=1,2) denote the coupling stiffness terms relating the classical plate deformation modes and transverse shear deformation modes. It is noted that A,B,D matrices could be different from those in Equation (7) due to possible nonzero $Y_{ij}$ (i=1,…,6;j=1,2).

Equations (10)–(12) form a system of 21 equations underpinning the Reissner–Mindlin model to be solved along with appropriate boundary conditions for 21 unknowns (five displacement variables, eight strain variables, and eight stress resultants, all of which are functions of $x_{1}$ and $x_{2}$ describing the 2D reference surface). Kinematics and kinetics remain the same no matter whether the structure is made of isotropic homogeneous materials such as metals or anisotropic heterogeneous materials such as composites. The only difference is that the plate/shell stiffness matrix in Equation (12) could be fully populated if the plate/shell is made of composites. Again, this model can be applied to model flat panels with arbitrary heterogeneity (e.g., laminates, sandwich panels with honeycomb cores, corrugated panels) as long as the thickness remains relatively small compared to the in-plane dimensions. The Reissner–Mindlin model governed by these equations has been implemented in many FEA codes which have plate/shell elements.

It is noted that the first-order shear deformation theory (FOSDT) is often used to model transverse shear deformation of composite plates/shells. It is actually a special application of the Reissner–Mindlin model to composite laminated plates/shells based on the particular assumptions that the transverse normal remains straight and rigid but not necessarily normal to the deformed reference surface.

It is noted that although the Reissner–Mindlin model was originally developed based on a set of a priori assumptions, such assumptions are not absolutely needed to derive this model if one can use a more advanced modeling technique such as VAM. Thus, the Reissner–Mindlin model presented here only refers to the model which has 21 field variables as functions of $x_{1},x_{2}$ and is governed by the 21 equations in Equations (10)–(12). The deformation and stress state of the structure along the thickness direction are not necessarily assumed a priori.

### 2.4. Euler–Bernoulli Beam Model

The kinematics of the Euler–Bernoulli beam model contains three displacements ${\bar{u}}_{1},{\bar{u}}_{2}$, ${\bar{u}}_{3}$, and one twist angle $\theta_{1}$. This model also features four strain variables including the axial strain $\epsilon_{11}$, the twist rate $\kappa_{11}$, and the curvatures $\kappa_{12},\kappa_{13}$ around $x_{2}$ and $x_{3}$, respectively. It is pointed out that the Euler–Bernoulli beam model is often formulated for bending only, and although some included extension, almost no formulation has also included torsion. In fact, torsion is also a fundamental deformation mode of a slender beam and the twist rate is of a similar order of magnitude of the bending curvatures for regular beams. Thus, we also include torsion into the Euler–Bernoulli beam model. Since the Euler–Bernoulli model is the first complete model developed in history, it is also called the classical beam model. The strain–displacement relations of the Euler–Bernoulli beam model are given as

(13) $$\epsilon_{11}=\frac{d{\bar{u}}_{1}}{dx_{1}},\;\kappa_{11}=\frac{d\theta_{1}}{dx_{1}},\;\kappa_{12}=-\frac{d^{2}{\bar{u}}_{3}}{dx_{1}^{2}},\;\kappa_{13}=\frac{d^{2}{\bar{u}}_{2}}{dx_{1}^{2}}$$

The kinetic variables of the Euler–Bernoulli beam model contain four stress resultants $F_{1},M_{1},M_{2},$$M_{3}$, with $F_{1}$ denoting the axial force and $M_{1},M_{2},M_{3}$ the moments about the three directions, governed by the following four equations of equilibrium:

(14) $$\begin{array}{cc} \; & \frac{dF_{1}}{dx_{1}}+p_{1}=0\\ \; & \frac{dM_{1}}{dx_{1}}+q_{1}=0\\ \; & \frac{d^{2}M_{2}}{dx_{1}^{2}}+p_{3}+\underline{\frac{dq_{2}}{dx_{1}}}=0\\ \; & \frac{d^{2}M_{3}}{dx_{1}^{2}}-p_{2}+\underline{\frac{dq_{3}}{dx_{1}}}=0 \end{array}$$

where $p_{1},p_{2},p_{3}$ are equivalent forces and $q_{1},q_{2},q_{3}$ are equivalent moments in three directions distributed along the reference line. It is noted here that most traditional formulations of the classical beam model only include the transverse pressure $p_{2}$ and $p_{3}$ as the distributed force. In fact, distributed forces could exist in three directions and distributed bending moment could also exist in three directions. These loads can be rigorously derived from the distributed body forces, tractions on the lateral surfaces. The two underlined terms in the last two equations of bending equilibrium are missing from almost all other formulations of the Euler–Bernoulli beam model.

The constitutive relations of the Euler–Bernoulli beam model can be expressed using the following four equations:

(15) $$\left\{\begin{array}{c} F_{1}\\ M_{1}\\ M_{2}\\ M_{3} \end{array}\right\}=\left[\begin{array}{cccc} C_{11}^{b} & C_{12}^{b} & C_{13}^{b} & C_{14}^{b}\\ C_{12}^{b} & C_{22}^{b} & C_{23}^{b} & C_{24}^{b}\\ C_{13}^{b} & C_{23}^{b} & C_{33}^{b} & C_{34}^{b}\\ C_{14}^{b} & C_{24}^{b} & C_{34}^{b} & C_{44}^{b} \end{array}\right]\left\{\begin{array}{c} \epsilon_{11}\\ \kappa_{11}\\ \kappa_{12}\\ \kappa_{13} \end{array}\right\}$$

This 4×4 symmetric matrix is the stiffness matrix and its inverse is the compliance matrix for the Euler–Bernoulli beam model.

In strength of materials, we have learned the following formulas for beams made of a single isotropic material:

(16) $$F_{1}=EA\epsilon_{11},\;M_{1}=GJ\kappa_{11},\;M_{2}=EI_{2}\kappa_{12},\;M_{3}=EI_{3}\kappa_{13}$$

with EA denoting the extension stiffness, GJ the torsion stiffness, and $EI_{2}$ and $EI_{3}$ the bending stiffness about $x_{2}$ and $x_{3}$, respectively. It is also noted here for a single isotropic material, *E* is the Young’s modulus, *G* is the shear modulus, *A* is the cross-sectional area, *J* is the torsion constant, and $I_{2}$ and $I_{3}$ are the area moments of inertia about $x_{2}$ and $x_{3}$, respectively. If the beam is made of multiple materials (possibly anisotropic), extension stiffness should not be interpreted as a single Young’s modulus times the cross-sectional area (i.e., E×A). In these cases, extension stiffness may still be referred to as EA but it should be calculated differently. Because each material has its own Young’s moduli along different directions, it is unlikely to obtain the extension stiffness using Young’s modulus of a real material multiplied by the area. The same comments apply to $GJ,EI_{2}$, and $EI_{3}$. The constitutive relations in Equation (16) can be written in the following matrix form as

(17) $$\left\{\begin{array}{c} F_{1}\\ M_{1}\\ M_{2}\\ M_{3} \end{array}\right\}=\left[\begin{array}{cccc} EA & 0 & 0 & 0\\ 0 & GJ & 0 & 0\\ 0 & 0 & EI_{2} & 0\\ 0 & 0 & 0 & EI_{3} \end{array}\right]\left\{\begin{array}{c} \epsilon_{11}\\ \kappa_{11}\\ \kappa_{12}\\ \kappa_{13} \end{array}\right\}$$

Equations (16) and (17) imply that the four fundamental deformation modes (extension, torsion, and bending in two directions) are completely decoupled. This is true for isotropic, homogeneous beams if the origin of the cross-sectional coordinates $x_{2},x_{3}$ is chosen to be at the tension center of the cross-section, and $x_{2}$ and $x_{3}$ are chosen to align with the principal bending directions of the beam. Otherwise, even if the beam is made of a single isotropic material, the four fundamental deformation modes will be coupled and some of the off-diagonal terms in the stiffness matrix in the right-hand side of Equation (17) will be nonzero. Therefore, the formulas in Equation (16) should not be used blindly. Proper offsets, including both positional and orientational offsets, must be used.

Equations (13)–(15) form a system of 12 equations underpinning the Euler–Bernoulli model to be solved along with appropriate boundary conditions for 12 unknowns (four displacement variables, four strain variables, and four stress resultants, all of which are functions of $x_{1}$ describing the beam reference line). Kinematics and kinetics remain the same no matter whether the structure is made of isotropic homogeneous materials such as metals or anisotropic heterogeneous materials such as composites. The only difference is that the beam stiffness matrix in Equation (15) could be fully populated if the beam is made of composites. These equations have been implemented in many FEA codes which have beam elements. Simple beam problems, particularly those with a uniform cross-section, can be solved analytically using the Euler–Bernoulli model.

It is noted that although the Euler–Bernoulli model was originally developed based on a set of a priori assumptions, including the cross-section being rigid in its own plane, remaining planar and perpendicular to the reference line, and the uniaxial stress assumption, such assumptions are not absolutely needed to derive this model if one can use a more advanced modeling technique such as VAM. Thus, the Euler–Bernoulli model presented here only refers to the model which has 12 field variables of $x_{1}$ governed by the 12 equations in Equations (13)–(15). In other words, the cross-section could be deformed, not necessarily perpendicular to the reference line, and all six stress components could exist. Actually, a beam-like structure which is modeled using the classical beam model may not even have clearly defined cross-sections, such as aircraft wings. As long as the structure is slender, a classical beam model can be used for the analysis. Furthermore, a slender structure can be analyzed using this model or any other model without specifically assuming that the cross-section should deform in a certain fashion or assuming that the structure should experience a certain type of stress state. Thus, calling a structure an Euler beam can cause unnecessary confusion and should be avoided.

### 2.5. Timoshenko Beam Model

When the cross-section of the beam is not very small compared to the length, or when the deformation cannot be fully captured using extension, twist, and two bending curvatures, the Euler–Bernoulli model is inadequate and a refined model is needed. A refined beam model beyond the Euler–Bernoulli model is the Timoshenko model. The kinematics of the Timoshenko beam model contains three displacements ${\bar{u}}_{1}$, ${\bar{u}}_{2}$, ${\bar{u}}_{3}$ and three rotations $\theta_{1}$, $\theta_{2}$, $\theta_{3}$. The Timoshenko beam model features the axial strain $\epsilon_{11}$, the transverse shear strains $\epsilon_{12},\epsilon_{13}$, the twist rate $\kappa_{11}$, and the curvatures $\kappa_{12},\kappa_{13}$. The strain–displacement relations are given as

(18) $$\begin{array}{cc} \; & \epsilon_{11}=\frac{d{\bar{u}}_{1}}{dx_{1}},\;2\epsilon_{12}=-\theta_{3}+\frac{d{\bar{u}}_{2}}{dx_{1}},\;2\epsilon_{13}=\theta_{2}+\frac{d{\bar{u}}_{3}}{dx_{1}}\\ \; & \kappa_{11}=\frac{d\theta_{1}}{dx_{1}},\;\kappa_{12}=\frac{d\theta_{2}}{dx_{1}},\;\kappa_{13}=\frac{d\theta_{3}}{dx_{1}} \end{array}$$

The kinetic variables of the Timoshenko beam model contain six stress resultants $F_{1}$, $F_{2}$, $F_{3}$, $M_{1}$, $M_{2}$, $M_{3}$ with $F_{1}$ denoting the axial force, $F_{2}$ and $F_{3}$ the transverse shear forces, and $M_{1},M_{2},M_{3}$ the moments about three directions, governed by the following six equations of equilibrium:

(19) $$\begin{array}{cc} \; & \frac{dF_{1}}{dx_{1}}+p_{1}=0\\ \; & \frac{dF_{2}}{dx_{1}}+p_{2}=0\\ \; & \frac{dF_{3}}{dx_{1}}+p_{3}=0\\ \; & \frac{dM_{1}}{dx_{1}}+q_{1}=0\\ \; & \frac{dM_{2}}{dx_{1}}-F_{3}+q_{2}=0\\ \; & \frac{dM_{3}}{dx_{1}}+F_{2}+q_{3}=0 \end{array}$$

The constitutive relations of the Timoshenko beam model can be expressed using the following six equations:

(20) $$\left\{\begin{array}{c} F_{1}\\ F_{2}\\ F_{3}\\ M_{1}\\ M_{2}\\ M_{3} \end{array}\right\}=\left[\begin{array}{cccccc} C_{11}^{b} & C_{12}^{b} & C_{13}^{b} & C_{14}^{b} & C_{15}^{b} & C_{16}^{b}\\ C_{12}^{b} & C_{22}^{b} & C_{23}^{b} & C_{24}^{b} & C_{25}^{b} & C_{26}^{b}\\ C_{13}^{b} & C_{23}^{b} & C_{33}^{b} & C_{34}^{b} & C_{35}^{b} & C_{36}^{b}\\ C_{14}^{b} & C_{24}^{b} & C_{34}^{b} & C_{44}^{b} & C_{45}^{b} & C_{46}^{b}\\ C_{15}^{b} & C_{25}^{b} & C_{35}^{b} & C_{45}^{b} & C_{55}^{b} & C_{56}^{b}\\ C_{16}^{b} & C_{26}^{b} & C_{36}^{b} & C_{46}^{b} & C_{56}^{b} & C_{66}^{b} \end{array}\right]\left\{\begin{array}{c} \epsilon_{11}\\ 2\epsilon_{12}\\ 2\epsilon_{13}\\ \kappa_{11}\\ \kappa_{12}\\ \kappa_{13} \end{array}\right\}$$

This 6×6 symmetric matrix is the stiffness matrix and its inverse is the compliance matrix of the Timoshenko model. It is noted that $C_{ij}^{b},i=1,2,3,4;j=1,2,3,4$ in this stiffness matrix could be different from those corresponding terms in Equation (15) due to possible couplings between the transverse shear modes and classical beam deformation modes.

From traditional structural mechanics, we know the following formulas for beams made of a single isotropic material:

(21) $$\begin{array}{cc} \; & F_{1}=EA\epsilon_{11},\;F_{2}=k_{2}GA\left(2\epsilon_{12}\right),\;F_{3}=k_{3}GA\left(2\epsilon_{13}\right)\\ \; & M_{1}=GJ\kappa_{11},\;M_{2}=EI_{2}\kappa_{12},\;M_{3}=EI_{3}\kappa_{13} \end{array}$$

with $k_{2}$ and $k_{3}$ the shear correction factors along $x_{2}$ and $x_{3}$, respectively, modifying the transverse shear stiffness GA due to the oversimplified constant strain assumption over the cross-section made in the traditional structural mechanics. These constitutive relations can be written in the following matrix form as

(22) $$\left\{\begin{array}{c} F_{1}\\ F_{2}\\ F_{3}\\ M_{1}\\ M_{2}\\ M_{3} \end{array}\right\}=\left[\begin{array}{cccccc} EA & 0 & 0 & 0 & 0 & 0\\ 0 & k_{2}GA & 0 & 0 & 0 & 0\\ 0 & 0 & k_{3}GA & 0 & 0 & 0\\ 0 & 0 & 0 & GJ & 0 & 0\\ 0 & 0 & 0 & 0 & EI_{2} & 0\\ 0 & 0 & 0 & 0 & 0 & EI_{3} \end{array}\right]\left\{\begin{array}{c} \epsilon_{11}\\ 2\epsilon_{12}\\ 2\epsilon_{13}\\ \kappa_{11}\\ \kappa_{12}\\ \kappa_{13} \end{array}\right\}$$

Equations (21) and (22) imply that the six fundamental deformation modes (extension, shear in two directions, torsion, and bending in two directions) are completely decoupled.

The diagonal stiffness matrix in Equation (22) is possible under four conditions: (i) the origin of the cross-sectional coordinates $x_{2},x_{3}$ is at the tension center of the cross-section, (ii) $x_{2},x_{3}$ align with the principal bending directions of the beam, (iii) the origin of $x_{2},x_{3}$ is at the shear center, and (iv) $x_{2},x_{3}$ align with the principal shear directions of the beam. Compared to the Euler–Bernoulli beam model, the diagonal stiffness matrix in Equation (17) only requires conditions (i) and (ii). All four conditions can be satisfied simultaneously only for idealized cross-sections, where the shear center is the same as the tension center and the principal shear directions the same as the principal bending directions. In other words, for most beams, even if the beam is made of a single isotropic material, a diagonal stiffness matrix for the Timoshenko model is not possible and some of the six fundamental deformation modes are coupled. Proper offsets, including both positional and orientational offsets, must be used before we can use the formulas in Equation (21).

Equations (18)–(20) form a system of 18 equations underpinning the Timoshenko beam model to be solved along with appropriate boundary conditions for 18 unknowns (six displacement variables, six strain variables, and six stress resultants, all of which are functions of $x_{1}$ describing the beam reference line). Kinematics and kinetics remain the same no matter whether the structure is made of isotropic homogeneous materials such as metals or anisotropic heterogeneous materials such as composites. The only difference is that the beam stiffness matrix in Equation (20) could be fully populated if the beam is made of composites. These equations have been implemented in many FEA codes which have beam elements. Simple beam problems, particularly those with a uniform cross-section, can be solved analytically using the Timoshenko model.

It is noted that FOSDT is also used to model transverse shear deformation of composite beams. It is actually a special application of the Timoshenko beam model to composite laminated beams based on the particular assumptions that the cross-section remains straight and rigid but not necessarily normal to the deformed reference line.

It is noted that although the Timoshenko beam model was originally developed based on a set of a priori assumptions, commonly called the Timoshenko assumptions, including the cross-section being rigid in its own plane, remaining in-plane during deformation, and experiencing special stress states, these assumptions are not absolutely needed to derive this model if one can use a more advanced modeling technique such as VAM. Thus, the Timoshenko beam model presented here only refers to the model which has 18 field functions of $x_{1}$ governed by the 18 equations in Equations (18)–(20). A beam-like structure can be analyzed using this model without specifically assuming that the cross-section should deform in a certain fashion or that the structure should experience a certain stress state. Thus, calling a structure a Timoshenko beam can cause unnecessary confusion and should be avoided.

### 2.6. Vlasov Beam Model

For thin-walled beams with open sections, the so-called constrained warping or nonuniform warping becomes significant. We need to refine the Euler–Bernoulli beam model into the Vlasov beam model, including the derivative of the twist rate as one of its strain variables. The displacement variables remain the same as the Euler–Bernoulli beam model, and the strain variables are $\epsilon_{11}$, $\kappa_{11}$, $\kappa_{12},\kappa_{13}$, and $\kappa_{11}^{\prime }$ with prime denotes the derivative with respect to $x_{1}$ with the following strain–displacement relations:

(23) $$\epsilon_{11}=\frac{d{\bar{u}}_{1}}{dx_{1}},\;\kappa_{11}=\frac{d\theta_{1}}{dx_{1}},\;\kappa_{12}=-\frac{d^{2}{\bar{u}}_{3}}{dx_{1}^{2}},\;\kappa_{13}=\frac{d^{2}{\bar{u}}_{2}}{dx_{1}^{2}},\;\kappa_{11}^{\prime }=\frac{d^{2}\theta_{1}}{dx_{1}^{2}}$$

The kinetic variables of the Vlasov beam model contain five stress resultants $F_{1}$, $M_{1}$, $M_{2}$, $M_{3}$, $M_{\omega }$ with $M_{\omega }$ denoting the bi-moment conjugate to $\kappa_{11}^{\prime }$. These five stress resultants are governed by the following four equations of equilibrium:

(24) $$\begin{array}{cc} \; & \frac{dF_{1}}{dx_{1}}+p_{1}=0\\ \; & \frac{dM_{1}}{dx_{1}}-\frac{d^{2}M_{\omega }}{dx_{1}^{2}}+q_{1}=0\\ \; & \frac{d^{2}M_{2}}{dx_{1}^{2}}+p_{3}+\frac{dq_{2}}{dx_{1}}=0\\ \; & \frac{d^{2}M_{3}}{dx_{1}^{2}}-p_{2}+\frac{dq_{3}}{dx_{1}}=0 \end{array}$$

The constitutive relations of the Vlasov beam model can be expressed using the following five equations:

(25) $$\left\{\begin{array}{c} F_{1}\\ M_{1}\\ M_{2}\\ M_{3}\\ M_{\omega } \end{array}\right\}=\left[\begin{array}{ccccc} C_{11}^{b} & C_{12}^{b} & C_{13}^{b} & C_{14}^{b} & C_{15}^{b}\\ C_{12}^{b} & C_{22}^{b} & C_{23}^{b} & C_{24}^{b} & C_{25}^{b}\\ C_{13}^{b} & C_{23}^{b} & C_{33}^{b} & C_{34}^{b} & C_{35}^{b}\\ C_{14}^{b} & C_{24}^{b} & C_{34}^{b} & C_{44}^{b} & C_{45}^{b}\\ C_{15}^{b} & C_{25}^{b} & C_{35}^{b} & C_{45}^{b} & C_{55}^{b} \end{array}\right]\left\{\begin{array}{c} \epsilon_{11}\\ \kappa_{11}\\ \kappa_{12}\\ \kappa_{13}\\ \kappa_{11}^{\prime } \end{array}\right\}$$

This 5×5 symmetric matrix is the stiffness matrix and its inverse is the compliance matrix for the Vlasov model. It is noted that $C_{ij}^{b},i=1,2,3,4;j=1,2,3,4$ in this stiffness matrix could be different from those corresponding terms in Equation (15) due to possible coupling between $\kappa_{11}^{\prime }$ and the classical beam deformation modes.

Equations (23)–(25) form a system of 14 equations to be solved along with appropriate boundary conditions for 14 unknowns (four displacement variables, five strain variables, and five stress resultants, all of which are functions of $x_{1}$ describing the beam reference line). Kinematics and kinetics remain the same no matter whether the structure is made of isotropic homogeneous materials such as metals or anisotropic heterogeneous materials such as composites. The only difference is that the beam stiffness matrix in Equation (25) could be fully populated if the beam is made of composites. These equations have been implemented in some FEA codes which have beam elements. Simple beam problems, particularly those with a uniform cross-section, can be solved analytically using the Vlasov model.

## 3. Common Methods for Deriving the Kirchhoff–Love Model for Composite Laminated Plates

Except the Cauchy continuum model, the other five models in Section 2 are also called structural models for beams, plates, and shells. These structural models are often derived from the Cauchy continuum model using three different methods, including the axiomatic method, the variational asymptotic method [14], and the formal asymptotic method [15]. Instead of commenting on what has been done using different methods, as is normal in other review papers, this paper will focus on illustrating the differences of these three methods to derive the Kirchhoff–Love model for composite laminated plates. This model is used for illustration because almost every student in mechanics of composite structures understands this model and it is the most extensively used model in the composites industry. 

### 3.1. Kirchhoff–Love Model Derived Using the Axiomatic Method

The axiomatic method is the most used method to model composite plates/beams/shells because it is relatively straightforward and intuitive. This method starts with some a priori assumptions for the displacement fields and/or the stress fields containing known functions of the thickness coordinate (for plates/shells) or the cross-sectional coordinates (for beams) and unknown functions of the large dimension(s). These assumed expressions are then usually substituted into a variational statement of the original 3D model (usually the Cauchy continuum model) to carry out the integration through the small dimensions to reduce the original 3D formulation into a one-dimensional (1D) formulation in terms of the beam axis for beams, and 2D formulation for plates/shells. This is essentially the application of the Kantorovich method [16] to composite structures. When this method is used to construct the Kirchhoff–Love model for composite laminates, it is also called CLT

To derive CLT for a composite laminate, the axiomatic method first introduces the so-called Kirchhoff–Love assumptions to express the displacement field as

(26) $$\begin{array}{cc} \; & u_{1}\left(x_{1},x_{2},x_{3}\right)={\bar{u}}_{1}\left(x_{1},x_{2}\right)-x_{3}{\bar{u}}_{3,1}\\ \; & u_{2}\left(x_{1},x_{2},x_{3}\right)={\bar{u}}_{2}\left(x_{1},x_{2}\right)-x_{3}{\bar{u}}_{3,2}\\ \; & u_{3}\left(x_{1},x_{2},x_{3}\right)={\bar{u}}_{3}\left(x_{1},x_{2}\right) \end{array}$$

With these expressions, the transverse normal line is assumed to be infinitely rigid along its own direction and remain straight and normal to the reference surface during deformation, which means additional constraints should be introduced to force the plate to behave in this fashion. In other words, with these assumptions, the structure is artificially made stiffer. Here, a comma denotes derivatives with respect to the in-plane coordinates, i.e., $f_{,\alpha }=\frac{\partial f}{\partial x_{\alpha }}$. Here and throughout the rest of this chapter, Greek indices α,β, …denote 1 or 2 and Latin indices denote 1, 2, or 3. Summation convention is applied to repeated indices.

Substituting the displacement assumptions in Equation (26) into the 3D strain definitions in Equation (1) using Equation (5), we obtain

(27) $$\begin{array}{cc} \varepsilon_{11}\left(x_{1},x_{2},x_{3}\right) & ={\bar{u}}_{1,1}-x_{3}{\bar{u}}_{3,11}=\epsilon_{11}+x_{3}\kappa_{11}\\ \varepsilon_{22}\left(x_{1},x_{2},x_{3}\right) & ={\bar{u}}_{2,2}-x_{3}{\bar{u}}_{3,22}=\epsilon_{22}+x_{3}\kappa_{22}\\ 2\varepsilon_{12}\left(x_{1},x_{2},x_{3}\right) & ={\bar{u}}_{1,2}+{\bar{u}}_{2,1}-2x_{3}{\bar{u}}_{3,12}=2\epsilon_{12}+x_{3}\kappa_{12}\\ \varepsilon_{13}\left(x_{1},x_{2},x_{3}\right) & =\varepsilon_{23}\left(x_{1},x_{2},x_{3}\right)=\varepsilon_{33}\left(x_{1},x_{2},x_{3}\right)=0 \end{array}$$

Because the transverse shear and normal strains ($\varepsilon_{13}$, $\varepsilon_{23}$, $\varepsilon_{33}$) vanish, the Kirchhoff–Love assumptions actually force a plane strain state to the composite laminates.

Next, the axiomatic method further adopts the plane stress assumption by assuming that only in-plane stresses ($\sigma_{11},\sigma_{12},\sigma_{22}$) exist in each layer and transverse shear and normal stresses ($\sigma_{13}$, $\sigma_{23}$, $\sigma_{33}$) vanish. This assumption agrees well with the exact solution if the thickness of the laminate is much smaller than the in-plane dimensions. Then, the 3D constitutive relations in Equation (3) are reduced to be

(28) $$\sigma_{e}=\left[\begin{array}{ccc} Q_{11} & Q_{12} & Q_{16}\\ Q_{12} & Q_{22} & Q_{26}\\ Q_{16} & Q_{26} & Q_{66} \end{array}\right]\varepsilon_{e}=Q\varepsilon_{e}$$

with *Q* as the *plane stress reduced stiffness matrix* and

$$\sigma_{e}=\left\{\begin{array}{c} \sigma_{11}\\ \sigma_{22}\\ \sigma_{12} \end{array}\right\},\;\varepsilon_{e}=\left\{\begin{array}{c} \varepsilon_{11}\\ \varepsilon_{22}\\ 2\varepsilon_{12} \end{array}\right\}$$

Next, the axiomatic method proceeds using either the Newtonian method or the variational method to derive the equilibrium equations. The more common approach is to use the variational method because it is more systematic and directly applicable to higher-order models.

The principle of virtual work of the plate structure can be stated as

(29) $$\int_{S}\delta U_{2D}dS=\delta W$$

with $U_{2D}$ as the 2D strain energy density defined over the reference surface, denoted using *S* and δ as the variation symbol in calculus of variations. The 2D strain energy density is the integration of the 3D strain energy density over the thickness such that

(30) $$U_{2D}=\frac{1}{2}\langle \langle \sigma_{e}^{T}\varepsilon_{e}\rangle \rangle$$

Double angle brackets indicate integration through the thickness, i.e., $\langle \langle f\rangle \rangle =\int_{h}fdx_{3}$.

The virtual work δW due to applied loads can be expressed as

(31) $$\delta W=\int_{S}\left(\langle \langle f_{i}\delta u_{i}\rangle \rangle +\beta_{i}\delta u_{i}\left(x_{1},x_{2},-\frac{h}{2}\right)+\tau_{i}\delta u_{i}\left(x_{1},x_{2},\frac{h}{2}\right)\right)\;dS+\int_{\Omega }\langle \langle \alpha_{i}\delta u_{i}\rangle \rangle d\Omega$$

Here, $f_{i}$ denotes the body force per unit volume, $\beta_{i}$ the traction on the bottom surface, $\tau_{i}$ the traction on the top surface, $\alpha_{i}$ the traction on the lateral surface, and Ω the boundary curve. Substituting the 3D displacement field expressed in Equation (26) into Equation (31), we have

(32) $$\delta W=\int_{S}\left(p_{i}\delta {\bar{u}}_{i}+q_{\alpha }\delta \Phi_{\alpha }\right)dS+\int_{\Omega }\left(P_{i}\delta {\bar{u}}_{i}+Q_{\alpha }\delta \Phi_{\alpha }\right)d\Omega$$

with

(33) $$\begin{array}{cc} p_{i} & =\langle \langle f_{i}\rangle \rangle +\beta_{i}+\tau_{i},\;q_{1}=\frac{h}{2}\left(\beta_{2}-\tau_{2}\right)-\langle \langle x_{3}f_{2}\rangle \rangle ,\;q_{2}=\frac{h}{2}\left(\tau_{1}-\beta_{1}\right)+\langle \langle x_{3}f_{1}\rangle \rangle \\ P_{i} & =\langle \langle \alpha_{i}\rangle \rangle ,\;Q_{1}=-\langle \langle x_{3}\alpha_{2}\rangle \rangle ,\;Q_{2}=\langle \langle x_{3}\alpha_{1}\rangle \rangle \end{array}$$

and $\Phi_{1}={\bar{u}}_{3,2}$ and $\Phi_{2}=-{\bar{u}}_{3,1}$ due to the axiomatic assumption that the transverse normal remains normal to the deformed reference surface. Here, we actually provided a systematic way to obtain the distributed forces $p_{i}\left(x_{1},x_{2}\right)$ and moments $q_{\alpha }\left(x_{1},x_{3}\right)$ along the reference surface, and the distributed forces $P_{i}$ and moments $Q_{\alpha }$ along the boundary curve in terms of the body forces and surface tractions applied on the original 3D structure.

Substituting the 3D stress field expressed in Equation (28) into Equation (30), we have

(34) $$U_{2D}=\frac{1}{2}\langle \langle \varepsilon_{e}^{T}\sigma_{e}\rangle \rangle =\frac{1}{2}\langle \langle \varepsilon_{e}^{T}Q\varepsilon_{e}\rangle \rangle$$

The in-plane strains $\varepsilon_{e}$ can be expressed in terms of the plate strains and curvatures as

$$\varepsilon_{e}=\epsilon +x_{3}\kappa$$

with $\epsilon =\lfloor \epsilon_{11}\;\;\epsilon_{22}\;\;2\epsilon_{12}\rfloor$ and $\kappa =\lfloor \kappa_{11}\;\;\kappa_{22}\;\;2\kappa_{12}\rfloor$. Substituting the above expressions into Equation (34), we have

(35) $$2U_{2D}={\left\{\begin{array}{c} \epsilon \\ \kappa \end{array}\right\}}^{T}\left[\begin{array}{cc} \langle \langle Q\rangle \rangle & \langle \langle x_{3}Q\rangle \rangle \\ \langle \langle x_{3}Q\rangle \rangle & \langle \langle x_{3}^{2}Q\rangle \rangle \end{array}\right]\left\{\begin{array}{c} \epsilon \\ \kappa \end{array}\right\}={\left\{\begin{array}{c} \epsilon \\ \kappa \end{array}\right\}}^{T}\left[\begin{array}{cc} A & B\\ B^{T} & D \end{array}\right]\left\{\begin{array}{c} \epsilon \\ \kappa \end{array}\right\}$$

with $A=\langle \langle Q\rangle \rangle ,B=\langle \langle x_{3}Q\rangle \rangle ,D=\langle \langle x_{3}^{2}Q\rangle \rangle$. It is noted that *B* is symmetric because *Q* is symmetric for composite laminates.

Carrying out the partial derivatives of $U_{2D}$ in Equation (35), we obtain

(36) $$\frac{\partial U_{2D}}{\partial \epsilon }=A\epsilon +B\kappa =N,\;\frac{\partial U_{2D}}{\partial \kappa }=B^{T}\epsilon +D\kappa =M$$

which is the same as the constitutive relations in Equation (7).

Substituting Equations (32) and (35) into Equation (29), following the usual procedure of calculus of variations, one can derive the equilibrium equations in Equation (6) as the Euler–Lagrange equations and corresponding boundary conditions. Equation (29) can also be used directly to formulate numerical solutions of the Kirchhoff–Love plate model.

The axiomatic method can be easily extended to develop other plate/shell models. One just needs to adjust the assumptions (different thickness functions, and unknown functions) for the displacements and/or the stresses, and decide whether these assumptions are applied to the entire structure or each individual layer. One can refer to many aforementioned review articles and the references listed in those articles to obtain a more comprehensive appreciation of the axiomatic method [3,4,5,6,7,8,9,10,11,12,13]. The following observations can be made regarding this method:

- This method must start with assumptions for the displacements and/or the stresses.
- These assumptions could be contradictory to each other. For example, CLT based on this method actually assumes both plane strain and plane stress, which cannot co-exist in general.
- This method does not provide a rational way to determine the loss of accuracy. For example, Kirchhoff assumptions make CLT stiffer than the original 3D model, while the plane stress assumptions make it softer. The combined effects of both are unknown in comparison to the original 3D model. The next refinement, FOSDT, corresponding to the Reissner–Mindlin model, derived by this method could actually be less accurate than CLT for some cases.
- This method has difficulty in directly satisfying the traction conditions on the top and bottom surfaces, and the continuity conditions for transverse shear and normal stresses on the interface between different layers. A mixed formulation using both displacements and stresses as variables is needed.
- Other than CLT and FOSDT, other models are different from what has been commonly implemented in commercial finite element packages, preventing their practical use in dealing with realistic structures using commercial finite element packages with readily available elements corresponding to the common engineering models presented in this paper.

### 3.2. Kirchhoff–Love Model Derived Using the Variational Asymptotic Method

As shown in the previous subsection, the 2D model corresponding to CLT is the Kirchhoff–Love plate model. Although CLT and the Kirchhoff–Love model were originally developed based on a set of ad hoc assumptions as aforementioned, such assumptions are not absolutely needed. In fact, one can use a more advanced modeling technique such as VAM to derive the Kirchhoff–Love model without these assumptions.

Considering laminates made of homogeneous layers, the linear elastic behavior is governed by the Cauchy continuum model. To construct a plate model, we need to first express the 3D displacements in terms of 2D plate displacements as:

(37) $$\begin{array}{cc} \; & u_{1}\left(x_{1},x_{2},x_{3}\right)={\bar{u}}_{1}\left(x_{1},x_{2}\right)-x_{3}{\bar{u}}_{3,1}+w_{1}\left(x_{1},x_{2},x_{3}\right)\\ \; & u_{2}\left(x_{1},x_{2},x_{3}\right)={\bar{u}}_{2}\left(x_{1},x_{2}\right)-x_{3}{\bar{u}}_{3,2}+w_{2}\left(x_{1},x_{2},x_{3}\right)\\ \; & u_{3}\left(x_{1},x_{2},x_{3}\right)={\bar{u}}_{3}\left(x_{1},x_{2}\right)+w_{3}\left(x_{1},x_{2},x_{3}\right) \end{array}$$

Here, $u_{i}\left(x_{1},x_{2},x_{3}\right)$ are 3D displacements, while ${\bar{u}}_{i}\left(x_{1},x_{2}\right)$ are plate displacements which are functions of $x_{1},x_{2}$ only. We also introduce 3D unknown fluctuating functions (also commonly called warping functions in structural mechanics) $w_{i}\left(x_{1},x_{2},x_{3}\right)$ to describe the information of 3D displacements which cannot be described by the simpler Kirchhoff–Love kinematics. Note that the displacement expressions in Equation (37) are not based on the Kirchhoff assumptions we have previously discussed. It can be considered as a change of variables to express the 3D displacements in terms of the displacement variables of the Kirchhoff–Love model and fluctuating functions. The Kirchhoff assumptions are equivalent to assuming no fluctuation (i.e., $w_{i}=0$). Since we consider that the Cauchy continuum model is our true model, we construct the Kirchhoff–Love plate model as an approximation to the true model. To this end, we need to define the plate displacements in terms of 3D displacements. A natural choice is:

(38) $$h{\bar{u}}_{3}\left(x_{1},x_{2}\right)=\langle \langle u_{3}\rangle \rangle ,\;h{\bar{u}}_{\alpha }\left(x_{1},x_{2}\right)=\langle \langle u_{\alpha }\left(x_{1},x_{2},x_{3}\right)\rangle \rangle +\langle \langle x_{3}\rangle \rangle {\bar{u}}_{3,\alpha }$$

which implies the following constraints on the fluctuating functions:

(39) $$\langle \langle w_{i}\rangle \rangle =0$$

Note that if the origin of the thickness coordinate is at the middle of the plate thickness, Equation (38) actually defines the plate displacements to be the average of the 3D displacements.

Then, the 3D strain field can be obtained as

$$\begin{array}{cc} \varepsilon_{11} & =\epsilon_{11}+x_{3}\kappa_{11}+w_{1,1}\\ 2\varepsilon_{12} & =2\epsilon_{12}+2x_{3}\kappa_{12}+w_{1,2}+w_{2,1}\\ \varepsilon_{22} & =\epsilon_{22}+x_{3}\kappa_{22}+w_{2,2}\\ 2\varepsilon_{13} & =w_{1,3}+w_{3,1}\\ 2\varepsilon_{23} & =w_{2,3}+w_{3,2}\\ \varepsilon_{33} & =w_{3,3} \end{array}$$

The 3D strain field can also be written in the following matrix form:

(40) $$\varepsilon_{e}=\epsilon +x_{3}\kappa +I_{\alpha }w_{\|,\alpha },\;\varepsilon_{t}=w^{\prime }+e_{\alpha }w_{3,\alpha }$$

with $w_{\|}={\left\lfloor w_{1}\;\;w_{2}\right\rfloor }^{T},w={\left\lfloor w_{1}\;\;w_{2}\;\;w_{3}\right\rfloor }^{T}$, $\varepsilon_{t}={\left\lfloor 2\varepsilon_{13}\;\;2\varepsilon_{23}\;\;\varepsilon_{33}\right\rfloor }^{T}$, and

(41) $$I_{1}=\left[\begin{array}{c} \begin{array}{cc} 1 & 0 \end{array}\\ \begin{array}{cc} 0 & 1 \end{array}\\ \begin{array}{cc} 0 & 0 \end{array} \end{array}\right],\;I_{2}=\left[\begin{array}{c} \begin{array}{cc} 0 & 0 \end{array}\\ \begin{array}{cc} 1 & 0 \end{array}\\ \begin{array}{cc} 0 & 1 \end{array} \end{array}\right],\;e_{1}=\left\{\begin{array}{c} 1\\ 0\\ 0 \end{array}\right\},\;e_{2}=\left\{\begin{array}{c} 0\\ 1\\ 0 \end{array}\right\}$$

The linear elastic problem of the composite laminate is governed by the variational statement in Equation (29) with the potential energy due to applied loads given in Equation (31) and double the 2D strain energy density expressed in the following form as

(42) $$2U=\langle \langle {\left\{\begin{array}{c} \varepsilon_{e}\\ \varepsilon_{t} \end{array}\right\}}^{T}\left[\begin{array}{cc} C_{e} & C_{et}\\ C_{et}^{T} & C_{t} \end{array}\right]\left\{\begin{array}{c} \varepsilon_{e}\\ \varepsilon_{t} \end{array}\right\}\rangle \rangle$$

In view of Equation (31), the work done by applied loads in the original 3D structure can be obtained as

(43) $$\begin{array}{cc} \delta W= & \int_{S}p_{i}\delta {\bar{u}}_{i}+q_{1}\delta {\bar{u}}_{3,2}-q_{2}\delta {\bar{u}}_{3,1}dS+\int_{\Omega }P_{i}\delta {\bar{u}}_{i}+Q_{1}\delta {\bar{u}}_{3,2}-Q_{2}\delta {\bar{u}}_{3,1}d\Omega \\ \; & +\int_{S}\left(\langle \langle f_{i}\delta w_{i}\rangle \rangle +\tau_{i}\delta w_{i}\left(x_{1},x_{2},\frac{h}{2}\right)+\beta_{i}\delta w_{i}\left(x_{1},x_{2},-\frac{h}{2}\right)\right)dS+\int_{\Omega }\langle \langle \alpha_{i}\delta w_{i}\rangle \rangle d\Omega \end{array}$$

Substituting Equations (42) and (43) into Equation (29), dropping smaller terms according to VAM, we have

(44) $$\begin{array}{cc} 0= & \int_{S}\delta \langle \langle {\left(\epsilon +x_{3}\kappa \right)}^{T}C_{e}\left(\epsilon +x_{3}\kappa \right)+w^{\prime T}C_{t}w^{\prime }+2{\left(\epsilon +x_{3}\kappa \right)}^{T}C_{et}w^{\prime }\rangle \rangle dS\\ & -\int_{S}p_{i}\delta {\bar{u}}_{i}+q_{1}\delta {\bar{u}}_{3,2}-q_{2}\delta {\bar{u}}_{3,1}dS-\int_{\Omega }P_{i}\delta {\bar{u}}_{i}+Q_{1}\delta {\bar{u}}_{3,2}-Q_{2}\delta {\bar{u}}_{3,1}d\Omega \end{array}$$

Minimizing this energy with respect to the fluctuating function $w_{i}$ along with the constraints in Equation (39), we reach the following Euler–Lagrange equations:

(45) $${\left({\left(\epsilon +x_{3}\kappa \right)}^{T}C_{et}+w^{\prime }C_{t}^{T}\right)}^{\prime }=\lambda$$

where λ contains three Lagrange multipliers enforcing the constraints in Equation (39). The boundary conditions on the top and bottom surfaces are

(46) $${\left(\epsilon +x_{3}\kappa \right)}^{T}C_{et}+w^{\prime }C_{t}=0$$

We can conclude that the above equation should be satisfied at every point through the thickness and solve for $w^{\prime }$ as

(47) $$w^{\prime }=-{\left(\epsilon +x_{3}\kappa \right)}^{T}C_{et}C_{t}^{-1}$$

$w_{i}$ can be solved by simply integrating through the thickness along with the interlaminar displacement continuity.

Substituting the solved fluctuating functions into Equation (44), we have

(48) $$2U_{0}=\langle \langle {\left(\epsilon +x_{3}\kappa \right)}^{T}Q\left(\epsilon +x_{3}\kappa \right)\rangle \rangle ={\left\{\begin{array}{c} \epsilon \\ \kappa \end{array}\right\}}^{T}\left[\begin{array}{cc} A & B\\ B^{T} & D \end{array}\right]\left\{\begin{array}{c} \epsilon \\ \kappa \end{array}\right\}$$

Replacing the strain energy term in Equation (44) with that in Equation (48), we will obtain a 2D variational statement exactly the same as that obtained in the previous subsection, which leads to the same equilibrium equations and boundary conditions.

This strain energy along with the work done by applied loads can be used to solve the 2D plate problem to obtain ${\bar{u}}_{i},\epsilon ,\kappa$. The 3D displacements can be obtained after we have solved for $w_{i}$.

(49) $$\begin{array}{cc} \; & u_{1}\left(x_{1},x_{2},x_{3}\right)={\bar{u}}_{1}\left(x_{1},x_{2}\right)-x_{3}{\bar{u}}_{3,1}+w_{1}\left(x_{1},x_{2},x_{3}\right)\\ \; & u_{2}\left(x_{1},x_{2},x_{3}\right)={\bar{u}}_{2}\left(x_{1},x_{2}\right)-x_{3}{\bar{u}}_{3,2}+w_{2}\left(x_{1},x_{2},x_{3}\right)\\ \; & u_{3}\left(x_{1},x_{2},x_{3}\right)={\bar{u}}_{3}\left(x_{1},x_{2}\right)+w_{3}\left(x_{1},x_{2},x_{3}\right) \end{array}$$

It is clear that the transverse normal does not remain rigid and normal according to the Kirchhoff–Love assumptions in CLT. Instead, the transverse normal can be deformed according to $w_{i}$.

The 3D strains can be obtained after neglecting the higher-order terms $w_{i,\alpha }$ which are not contributing to the strain energy captured by the Kirchhoff–Love plate model:

(50) $$\varepsilon_{e}=\epsilon +x_{3}\kappa ,\;\varepsilon_{t}=-{\left(\epsilon +x_{3}\kappa \right)}^{T}C_{et}C_{t}^{-1}$$

Clearly, the strain field is not in-plane as is traditionally assumed using the Kirchhoff assumptions in CLT. Instead, transverse shear and normal strains could both exist.

The 3D stresses can be obtained by directly using the above strain field along with Hooke’s law of the original 3D elasticity theory as

(51) $$\sigma_{e}=Q\left(\epsilon +x_{3}\kappa \right)$$

and transverse shear and normal stresses vanish. It can be observed that the Kirchhoff–Love model derived using VAM exhibits a plane stress state. However, this is not assumed a priori as in the axiomatic method but derived by using VAM instead.

Comparing to the CLT derived using the axiomatic method, we observe that VAM does not require a priori assumptions such as the Kirchhoff–Love assumptions and plane stress assumption. The displacement field, strain field, and stress field are completely compatible with each other. The CLT derived by VAM features layerwise quadratic distributions of the 3D displacements, layerwise linear distributions for the transverse shear, and normal strains. As shown in [17,18,19], VAM can be used to derive the Reissner–Mindlin model with the following features:

- The transverse displacement is a piecewise quadratic function of $x_{3}$, and the in-plane displacements are piecewise cubic functions of $x_{3}$.
- The in-plane stresses and strains are piecewise cubic functions of $x_{3}$, and the transverse shear stresses and strains are piecewise quadratic functions of $x_{3}$, and the transverse normal stress and strain are piecewise cubic functions of $x_{3}$.
- The 3D displacements and stresses satisfy both the displacement and traction continuity on the interfaces.
- The 3D stresses satisfy traction conditions on the top and bottom surfaces.
- The 3D stresses satisfy the first two equations of the 3D equilibrium asymptotically up to the second order and the third equation in the sense of minimal energy loss in a Reissner–Mindlin model.

The accuracy of the Reissner–Mindlin model derived using VAM is almost the same as fourth-order zigzag theories or lower-order layerwise theories for regular laminates. Such a theory can easily satisfy many of the needs mentioned in [13] for refined theories beyond the Kirchhoff–Love model and the Reissner–Mindlin model if such models were derived using the axiomatic method.

VAM was originally invented by Berdichevsky [20] and applied and popularized by Hodges to composite structures [18,21,22,23,24,25,26,27,28,29,30,31,32,33,34,35,36,37,38,39,40,41,42,43,44,45,46,47,48,49,50,51,52,53,54,55,56]. Another unique feature of the work by Hodges and his co-workers is that those structure models they developed are geometrically exact, which is far beyond the von Karman-type nonlinearity commonly used in those models developed using the axiomatic method.

Like the two other methods, VAM also has some drawbacks. First, it relies on asymptotical analysis of functionals, which is less intuitive than the axiomatic method and FAM. Second, the second-order asymptotically correct energy is not in the form of a Reissner–Mindlin model; some transformation, which might lose accuracy, needs to be employed. Lastly, it is becoming much harder to derive higher-order models. Fortunately, higher-order models are not frequently used in practical structural design and analysis.

### 3.3. Kirchhoff–Love Model Derived Using the Formal Asymptotic Method

The formal asymptotic method (FAM) is another method commonly used to derive models for composite structures [15,57,58,59,60,61]. The basic idea is to expand all the field variables asymptotically and solve the governing different equations or the corresponding variational statement asymptotically according to different orders. The asymptotic expansion is performed in terms of the small parameter (ζ), which is used to describe the smallness of a certain structural dimension such as the thickness with respect to the in-plane dimensions for composite laminated plates. In particular, we scale the thickness coordinate for a composite laminated plate so that

(52) $$y_{3}=\frac{x_{3}}{\zeta },\;\frac{\partial f}{\partial x_{3}}=\frac{1}{\zeta }\frac{\partial f}{\partial y_{3}}$$

FAM starts with the assumption that the displacement field can be expanded into an asymptotic series such that

(53) $$u_{i}=u_{i}^{\left(0\right)}+\zeta \;u_{i}^{\left(1\right)}\left(x,y\right)+\zeta^{2}u_{i}^{\left(2\right)}\left(x,y\right)+O\left(\zeta^{3}\right)$$

Here, FAM assumes $u_{\alpha }^{\left(0\right)}=0,u_{3}^{\left(0\right)}=v_{3}^{\left(0\right)}\left(x_{1},x_{2}\right)$. It is noted that the superscripts here indicate the asymptotic order of the corresponding quantities, not powers.

Substituting Equation (53) into Equation (1) and considering Equation (52), we obtain

(54) $$\varepsilon_{ij}=\varepsilon_{ij}^{\left(0\right)}+\zeta \;\varepsilon_{ij}^{\left(1\right)}+O\left(\zeta^{2}\right)$$

with

(55) $$\begin{array}{cc} \varepsilon_{\alpha \beta }^{\left(0\right)} & =0,\;\varepsilon_{\alpha 3}^{\left(0\right)}=\frac{1}{2}\left(v_{3,\alpha }^{\left(0\right)}+\frac{\partial u_{\alpha }^{\left(1\right)}}{\partial y_{3}}\right),\;\varepsilon_{33}^{\left(0\right)}=\frac{\partial u_{3}^{\left(1\right)}}{\partial y_{3}} \end{array}$$

(56) $$\begin{array}{cc} \varepsilon_{\alpha \beta }^{\left(1\right)} & =\frac{1}{2}\left(u_{\alpha ,\beta }^{\left(1\right)}+u_{\beta ,\alpha }^{\left(1\right)}\right),\;\varepsilon_{\alpha 3}^{\left(1\right)}=\frac{1}{2}\left(u_{3,\alpha }^{\left(1\right)}+\frac{\partial u_{\alpha }^{\left(2\right)}}{\partial y_{3}}\right),\;\varepsilon_{33}^{\left(1\right)}=\frac{\partial u_{3}^{\left(2\right)}}{\partial y_{3}} \end{array}$$

FAM continues to assume that there is no need to expand the elasticity tensor $C_{ijkl}$ into an asymptotic series, which implies that we can obtain the following:

(57) $$\sigma_{ij}=\sigma_{ij}^{\left(0\right)}+\zeta \;\sigma_{ij}^{\left(1\right)}+O\left(\zeta^{2}\right)$$

with

$$\sigma_{ij}^{\left(0\right)}=C_{ijkl}\varepsilon_{kl}^{\left(0\right)},\;\sigma_{ij}^{\left(1\right)}=C_{ijkl}\varepsilon_{kl}^{{(}^{1})}$$

Substituting the asymptotic expansion into Equation (30), we can obtain the asymptotic expansion of the 2D strain energy density up to the second order as

(58) $$U_{2D}=\zeta U_{2D}^{\left(0\right)}+\zeta^{2}U_{2D}^{\left(1\right)}+\zeta^{3}U_{2D}^{\left(2\right)}+O\left(\zeta^{4}\right)$$

with

(59) $$\begin{array}{cc} U_{2D}^{\left(0\right)} & =\frac{1}{2}\langle {\left(\sigma^{\left(0\right)}\right)}^{T}\varepsilon^{\left(0\right)}\rangle \end{array}$$

(60) $$\begin{array}{cc} U_{2D}^{\left(1\right)} & =\frac{1}{2}\langle {\left(\sigma^{\left(1\right)}\right)}^{T}\varepsilon^{\left(0\right)}+{\left(\sigma^{\left(0\right)}\right)}^{T}\varepsilon^{\left(1\right)}\rangle \end{array}$$

(61) $$\begin{array}{cc} U_{2D}^{\left(2\right)} & =\frac{1}{2}\langle {\left(\sigma^{\left(1\right)}\right)}^{T}\varepsilon^{\left(1\right)}+{\left(\sigma^{\left(0\right)}\right)}^{T}\varepsilon^{\left(2\right)}+{\left(\sigma^{\left(2\right)}\right)}^{T}\varepsilon^{\left(0\right)}\rangle \end{array}$$

Here, $\langle \langle \cdot \rangle \rangle =\zeta \int \cdot dy_{3}=\zeta \langle \cdot \rangle$.

To expand the virtual work asymptotically, FAM assumes the following asymptotic orders for body forces and tractions:

$$f_{\alpha }∼\alpha_{\alpha }∼\zeta ,\;f_{3}∼\alpha_{3}∼\zeta^{2},\;\beta_{\alpha }∼\tau_{\alpha }∼\zeta^{2},\;\beta_{3}∼\tau_{3}∼\zeta^{3}$$

The virtual work can be written as

(62) $$\begin{array}{cc} \delta W= & \int_{S}\left(\langle \langle f_{\alpha }\delta u_{\alpha }^{\left(1\right)}+f_{3}\delta v_{3}^{\left(0\right)}\rangle \rangle +\beta_{\alpha }\delta u_{\alpha }^{\left(1\right)}\left(-\frac{h}{2}\right)+\left(\beta_{3}+\tau_{3}\right)\delta v_{3}^{\left(0\right)}+\tau_{\alpha }\delta u_{\alpha }^{\left(1\right)}\left(\frac{h}{2}\right)\right)\;dS+\\ & \int_{\Omega }\langle \langle \alpha_{\alpha }\delta u_{\alpha }^{\left(1\right)}+\alpha_{3}\delta v_{3}^{\left(0\right)}\rangle \rangle d\Omega +O\left(\zeta^{4}\right) \end{array}$$

The first-order variational statement is $\delta U_{2D}^{\left(0\right)}=0$, which can be used to conclude

(63) $$\varepsilon^{\left(0\right)}=0,\;\sigma^{\left(0\right)}=0$$

One can obtain $u_{i}^{\left(1\right)}$ as

(64) $$u_{\alpha }^{\left(1\right)}=v_{\alpha }^{\left(1\right)}\left(x_{1},x_{2}\right)-y_{3}v_{3,\alpha }^{\left(0\right)},\;u_{3}^{\left(1\right)}=v_{3}^{\left(1\right)}\left(x_{1},x_{2}\right)$$

which is the same as those in Equation (26) given by the Kirchhoff–Love assumptions after some simple changes of variables.

Based on the first-order solution in Equation (64), we can write out the second-order solution as

(65) $$u_{\alpha }^{\left(2\right)}=v_{\alpha }^{\left(2\right)}\left(x_{1},x_{2}\right)-y_{3}v_{3,\alpha }^{\left(1\right)}+w_{\alpha }\left(x_{1},x_{2},y_{3}\right),\;u_{3}^{\left(2\right)}=v_{3}^{\left(2\right)}\left(x_{1},x_{2}\right)+w_{3}\left(x_{1},x_{2},y_{3}\right)$$

Substituting Equation (65) into Equation (56), we obtain the first-order strains as

(66) $$\begin{array}{cc} \varepsilon_{\alpha \beta }^{\left(1\right)} & =\frac{1}{2}\left(v_{\alpha ,\beta }^{\left(1\right)}+v_{\beta ,\alpha }^{\left(1\right)}\right)-y_{3}v_{3,\alpha \beta }^{\left(0\right)},\;\varepsilon_{\alpha 3}^{\left(1\right)}=\frac{1}{2}\frac{\partial w_{\alpha }}{\partial y_{3}},\;\varepsilon_{33}^{\left(1\right)}=\frac{\partial w_{3}}{\partial y_{3}} \end{array}$$

In view of Equation (63), we can conclude

$$U_{2D}^{\left(1\right)}=0,\;U_{2D}^{\left(2\right)}=\frac{1}{2}\langle {\left(\sigma^{\left(1\right)}\right)}^{T}\varepsilon^{\left(1\right)}\rangle$$

If we let

$${\bar{u}}_{\alpha }=v_{\alpha }^{\left(1\right)},\;{\bar{u}}_{3}=v_{3}^{\left(0\right)}$$

and

$$\epsilon_{\alpha \beta }=\frac{1}{2}\left(v_{\alpha ,\beta }^{\left(1\right)}+v_{\beta ,\alpha }^{\left(1\right)}\right),\;\kappa_{\alpha \beta }=-v_{3,\alpha \beta }^{\left(0\right)}$$

then the second order of the virtual work in Equation (62) is exactly the same as Equation (32), and the variation in the total potential energy of the second order is exactly the same as that derived by VAM in Equation (44). This further implies that the warping functions solved from FAM are exactly the same as those solved by VAM given in Equation (47). The corresponding macroscopic plate model, CLT, remains the same. Since, at this stage, we have only solved $v_{\alpha }^{\left(1\right)},v_{3}^{\left(0\right)}$, the 3D displacements, strains, and stresses can be computed in the same way if we do not include the higher-order 2D functions $v_{\alpha }^{\left(2\right)},v_{3}^{\left(1\right)}$ to be solved later in the macroscopic 2D plate model of the next asymptotic expansion. To reproduce the expressions exactly the same, we need to set ζ=1, and $y_{3}$ becomes $x_{3}$.

Although both VAM and FAM provide the same Kirchhoff–Love model, we can observe that FAM requires more careful set up of the asymptotic order and lengthier derivation. Also, it introduces more 2D functions to construct the Kirchhoff–Love model and there are no relations of the 2D functions for different orders. In fact, each asymptotic expansion corresponds to a macroscopic plate model with five new 2D functions which can be solved recursively, as shown in Ref. [15]. These models are not compatible with those plate/shell elements in off-the-shelf commercial FEA codes such as Abaqus, Ansys, or Nastran.

## 4. Future Directions for Modeling of Composite Structures

The author believes that it is unnecessary to go beyond the classical structural models as long as the models are constructed without a priori assumptions. These structural models constructed by VAM are sufficient for most engineering practices as long as the basic requirements are satisfied, such as the plate/shell being thin and the beam being slender. To obtain better predictions than these models usually requires more sophisticated models with not much computational savings in comparison to the original 3D model, particularly in view of the fact that we need to develop special-purpose finite element codes for those sophisticated models. The model setup time and loss of versatility in modeling realistic structures quickly outweigh the insignificant computing speed-up by those models which are more sophisticated than the classical models introduced in this paper.

As far as the future directions are concerned, the global structural analysis using the classical structural models is already implemented in many commercial finite element packages. Not much research to be carried out there; instead, we need to convince those codes to open up to accept fully populated stiffness matrices, or tangent stiffness matrix for nonlinear behavior.

Since VAM does not rely on a priori assumptions, which are usually heavily dependent on the particular structure, such as laminates, these approach can be easily extended to other types of structures.

Many linear problems have been worked out by Hodges and his co-workers and the advantages of such models are clearly shown [18,21,22,23,24,25,26,27,28,29,30,31,32,33,34,35,36,37,38,39,40,41,42,43,44,45,46,47,48,49,50,51,52,53,54,55,56]. It is time to explore using VAM to solve nonlinear static or dynamic problems such as local buckling, damage and failure analysis, simulation of manufacturing processes, multiscale design, dynamic properties for anisotropic heterogeneous materials and structures, and transport properties needed for composites manufacturing. All these directions are actively pursued by the author and his co-workers.

## 5. Conclusions

Modeling of composite structures is reviewed in terms of the modeling methods, including the axiomatic methods VAM, and FAM. Their differences were highlighted through deriving CLT. The advantages of VAM was compared with respect to the axiomatic method and VAM. VAM was invented by Berdichevsky and was applied and popularized by Hodges and his co-workers. Because of his work, a special area of research, constitutive modeling of structures, is established and should be more vigorously pursued. Classical structural models constructed by VAM are better than most high-order equivalent single-layer models, zigzag models, or layerwise models developed using the axiomatic methods. The practical advantages of classical structural models already present in commercial finite element packages cannot be emphasized enough because those are the tools used by engineers everyday. Several promising directions for future research are pointed out.

## Data Availability

No new data were created.
